# Association between *IL*-*18* polymorphisms, serum levels, and HBV-related hepatocellular carcinoma in a Chinese population: a retrospective case–control study

**DOI:** 10.1186/s12935-015-0223-z

**Published:** 2015-07-25

**Authors:** Jingui Bao, Yu Lu, Yan Deng, Chengzhi Rong, Yanqiong Liu, Xiuli Huang, Liuying Song, Shan Li, Xue Qin

**Affiliations:** Department of Clinical Laboratory, First Affiliated Hospital of Guangxi Medical University, Nanning, 530021 Guangxi China

**Keywords:** Hepatocellular carcinoma, Interleukin-18, Polymorphisms

## Abstract

**Background:**

*Interleukin (IL)*-*18* gene polymorphisms have been found to play multiple roles in various diseases. However, studies focused on its involvement in hepatocellular carcinoma (HCC) remains controversial, and no much study has taken IL-18 serum levels into consideration. This study investigates the association between *IL*-*18* polymorphisms and risk of hepatitis B virus-related HCC and their impact on serum IL-18 serum levels.

**Methods:**

A total of 153 patients and 165 healthy controls were enrolled in this study. Polymorphisms at positions −607C/A and −137G/C in the *IL*-*18* gene were determined using the polymerase chain reaction-restriction fragment length polymorphism method. Serum IL-18 levels were determined with an ELISA kit.

**Results:**

No relationship was found between the −607C/A polymorphism and an individual’s susceptibility to HCC. For the −137G/C polymorphism, the GC genotype and C allele were found to be significantly associated with decreased HCC risk (OR 0.506, 95% CI 0.290–0.882, *P* = 0.016 and OR 0.520, 95% CI 0.332–0.814, *P* = 0.004, respectively). The A^−607^C^−137^ haplotype was also associated with a significant decreased risk of HCC (OR 0.495, 95% CI 0.294–0.834, *P* = 0.007). Serum IL-18 levels were found to be significantly lower in HCC patients compared to the control group in both the overall population and subjects with the different SNPs. Further, no association was found between serum IL-18 levels and the different genotypes within the same SNP.

**Conclusion:**

These findings suggest that the −137G/C SNP in *IL*-*18* may be a protective factor against HCC. Nevertheless, none of the studied SNPs was associated with the expression of IL-18.

## Background

Hepatitis B virus (HBV) infection is highly prevalent and one of the major causes of morbidity and death worldwide. Globally, two billion people have been reported to be infected with HBV, of which approximately one-fifth (about 350–400 million) are chronic carriers; further, tens of millions of new HBV cases occur annually [1]. The outcome of chronic HBV infection is highly variable, ranging from an asymptomatic carrier state with a normal liver histology to persistent carriers with severe and chronic liver diseases such as cirrhosis and hepatocellular carcinoma (HCC) [2]. HCC is the fifth most frequent cancer in men and the seventh in women worldwide [3], and ranks as the second most common malignancy in China, especially in Southern Guangxi [4]. Among individuals persistently infected with HBV, one to three in every ten will develop liver cirrhosis and HCC [5]. Such highly variable outcomes may be generally attributed to viral factors such as HBV infection [6] and environmental factors such as dietary aflatoxin B1 exposure [7], along with an individual’s immunological status and genetic background [8]. Further, according to Peng et al. [9], the morbidity of HCC can be distinct in individuals with the same exposure, suggesting that host immunological status and genetic factors may play important roles in the development of HBV-related HCC.

Interleukin-18 (IL-18), a novel proinflammatory cytokine belonging to the IL-1 cytokine super-family, is mainly produced by activated macrophages and can mediate both innate and adaptive immunity [10, 11]. It was initially recognized as an interferon-γ (IFN-γ)-inducing factor [12] and has a wide range of functions, including induction of the synthesis of IFN-γ in T cells and natural killer cells through synergistic action with IL-12 and IL-10, respectively [13–15], promotion of Th1-type immune responses, and enhancement of the proliferative response and cytokine production of activated T cells [16]. Recently, this cytokine was found to play various roles in chronic inflammation [17] and autoimmune diseases, as well as in numerous infectious diseases [18–20]. Further, IL-18 has been linked to acute liver injury [21]. As HCC is a typical inflammation-related cancer, these biologic effects of IL-18 might thus be implicated with HCC development, suggesting that abnormal expression of IL-18 could be associated with the pathogenesis of this disease.

The human *IL*-*18* gene is located on chromosome 11q22.2-22.3 and consists of six exons and five introns [22]. So far, three single nucleotide polymorphisms (SNPs) in the promoter region of the *IL*-*18* gene have been elucidated, namely −656G/T (rs1946518), −607C/A (rs1946519), and −137G/C (rs187238) [23]. A change from the C to the A allele at position −607C/A and a change from the G to the C allele at position −137G/C in the promoter region were predicted to be the nuclear factor binding sites for the cAMP responsive element binding protein and H4TF-1 nuclear factor, respectively. Furthermore, polymorphisms of the two sites have been associated with *IL*-*18* gene promoter transcription activity, which can influence the expression of IL-18 and, potentially, of IFN-γ [23, 24]; these allelic changes may be the underlying mechanism of *IL*-*18* involvement in various diseases. To date, *IL*-*18* polymorphisms have been proved associated with various diseases, including chronic HBV infection [17], systemic lupus erythematosus [25], breast cancer [26], oral cancer [27, 28], thyroid cancer [29], colorectal cancer [30], and bladder cancer [31]. However, results focused on the effect of *IL*-*18* polymorphisms or IL-18 expression on the risk of HBV-related HCC remains controversial [32–34], and no much study has taken IL-18 serum levels into consideration. The present study was performed in order to further investigate the possible role of *IL*-*18* −607C/A and −137G/C polymorphisms on the susceptibility to HBV-related HCC and their impact on serum IL-18 serum levels. This study compares *IL*-*18* polymorphisms and IL-18 serum levels between HBV-related HCC patients and healthy controls from Guangxi, China, a region with a high HBV prevalence.

## Methods

### Patients and controls

The study includes 153 HBV-related HCC patients and 165 healthy controls, of which were estimated using Quanto software (version 1.2.4). We assumed that the prevalence of the−607C/A (rs1946519) AA genotype in the control group was 38.1% (according to HapMap Project dbSNP database: http://www.ncbi.nlm.nih.gov/snp/, the HCB population), and estimated OR was 2.0 with probability of α = 0.05, β = 0.1 and matched case–control design. According to the parameters set, the calculation result was 136, a sample size with 153 cases and 165 controls was therefore considered had enough power to evaluate the risk of the IL-18 genetic variation on HCC development. All participants were recruited from the First Affiliated Hospital of Guangxi Medical University between May and December 2013. Inclusion criteria for the HCC group were as follows: (1) All patients were seropositive to hepatitis B surface antigen, HBV core antibody, hepatitis Be antigen, or hepatitis Be antibody for at least 6 months; (2) newly diagnosed HCC patients with positive cytologic or pathologic findings for HCC; and (3) without other hepatitis virus infections such as hepatitis C or hepatitis E. The control age-, sex- and ethnic- matched subjects were randomly selected from the same hospital and confirmed to be HBV free and without any malignancy or other serious illness. Other demographic and laboratory data such as smoking habits, alcohol consumption (both defined as never and current), level of alanine aminotransferase (ALT), and aspartate aminotransferase (AST) were obtained using electronic medical records. Informed consent for genetic analysis was obtained from all participants. The study was approved by the ethics committee of the First Affiliated Hospital of Guangxi Medical University.

### Determination of the *IL*-*18* genotypes

About 2 mL peripheral blood samples in ethylenediaminetetraacetic acid (EDTA)-coated vials were collected from all of the participants and stored at −80°C until use. Genomic DNA was extracted using a QIAamp DNA blood mini kit (QIAGEN GmbH, Hilden, Germany). Amplification of the target DNA was performed by polymerase chain reaction (PCR). *IL*-*18* gene polymorphisms at positions −607C/A and −137G/C were identified using PCR-restriction fragment length polymorphism. The primer sequences, annealing temperature, restriction enzymes used, and length of the PCR products are shown in Table 1. To confirm the genotyping results, 32 samples (about 10%) were randomly selected and directly sequenced by Sangon Biotech Company with an ABI Prism 3100 (Shanghai, China). A 100% concordance rate was achieved.

**Table 1:** Primer sequences and reaction conditions for genotyping *IL*-*18* polymorphisms

Polymorphisms	Primer sequence	Annealing temperature	Restriction enzyme	Product size (bp)
−607C/A	F:5′-TCAGTGGAACAGGAGTCCAT-3′	50°C	DraI	AA: 109 + 41
	R:5′-GCAGAAAGTGTAAAAATTTTT-3′			CC: 150
				AC: 109 + 41 + 150
−137G/C	F:5′-AGGTGCTTTCTTAAAGTCAGA-3′	50°C	HinfI	GG: 107 + 42
	R:5′-AATATCACRATTTTCATGGAA-3′			CC: 149
				GC: 107 + 42 + 149

### Serum IL-18 levels

Serum samples without any anticoagulation were collected for all cases and controls. Following the obtainment of blood specimens, the serum was allowed to clot for 30 min at room temperature before centrifugation at 2,000 rpm for 10 min at 4°C. The serum was then isolated and stored at −80°C until further use. A sandwich ELISA kit (Human Interleukin 18 ELISA Kit, Sangon Biotech Company, Shanghai, China) was used, according to the manufacturer’s instructions, to determine IL-18 serum levels for all subjects. The minimum level of detection for IL-18 was 15 ng/L. Each sample was assayed in duplicate and the intra-assay coefficient of variation was 7.5%.

### Statistical analysis

We used the Student’s *t* test and the χ^2^ test to evaluate the distribution of the general demographic and clinical features between cases and controls. The Hardy-Weinberg equilibrium (HWE) was tested via a goodness-of-fit χ^2^ test with 1° of freedom to compare the observed genotype frequencies among the subjects with the expected genotype frequencies. Allele frequency and genotype distribution of *IL*-*18* gene polymorphisms were compared using the χ^2^ test and Fisher’s exact test, as appropriate. The association between the genotypes and HCC risk was estimated by odds ratios (ORs) with corresponding 95% confidence intervals (CIs) calculated using the binary logistic regression model and were further adjusted for age, sex, smoking, and alcohol consumption status. Haplotypes and their frequencies were also estimated based on a Bayesian algorithm with the Phase program [35]. For serum IL-18 levels, means and standard deviations (SD) were used to express the data if variables were normally distributed, if not, they were described as median and interquartile range. One-way ANOVA or the Kruskal–Wallis test were used to examine the difference of serum IL-18 levels in different genotypes with the same group subjects, in situations of normal or skewed distribution, respectively; if significant, these were followed by the Student–Newman–Keuls test. The Student’s *t*-test or Mann–Whitney U-test were used to examine the difference in serum IL-18 levels between the two groups among individuals with the same genotype, in situations of normal or skewed distribution, respectively. All statistical analyses were performed using the statistical software package SPSS 16.0 (SPSS Inc., Chicago, IL, USA), with two-sided *P* values <0.05 being considered as statistically significant.

## Results

### Clinical characteristics of the study subjects

The demographic and clinical characteristics of the cases and control subjects are shown in Table 2. The mean ages (SD) in the control and patient groups were 47.37 ± 11.55 and 49.18 ± 11.36, respectively, with no significant difference between them. Further, there were no significant differences in sex, smoking, or alcohol consumption status, suggesting that the case data were comparable with those of the controls (all *P* > 0.05). However, the values of ALT and AST were significantly higher in HCC patients than in controls (both p < 0.001)

**Table 2:** Clinical characteristic of the study subjects

Variable	Cases, n = 153 (%)	Controls, n = 165 (%)	*P*
Age (mean ± SD)	49.18 ± 11.36	47.37 ± 11.55	0.836
Sex			
Male	134 (87.6)	143 (86.7)	0.808
Female	19 (12.4)	22 (13.3)	
Ethnic(Han/Zhuang)	92/61	102/63	0.758
Alcohol consumption			
No	112 (73.2)	120 (72.7)	0.924
Yes	41 (26.8)	45 (27.3)	
Cigarette smoking			
No	118 (77.1)	125 (75.8)	0.774
Yes	35 (22.9)	40 (24.2)	
ALT(mean ± SD)	56.42 ± 28.04	29.62 ± 6.85	0.001
AST(mean ± SD)	55.29 ± 28.47	30.12 ± 6.65	0.001

*ALT* alanine aminotransferase, *AST* aspartate aminotransferase, *SD* standard deviation.

### Association between −607C/A SNP and HBV-related HCC risk

The genotype and allele frequencies of *IL*-*18* −607C/A polymorphisms in the HBV-related HCC patients and healthy controls are shown in Table 3. According to the HWE test, the genotype distribution of this SNP in controls were within HWE (*P* = 0.322). The frequencies of the AA, AC, and CC genotypes of −607C/A were 24.8, 46.1, and 29.1%, respectively, in healthy controls and 24.2, 47.7, and 28.1%, respectively, in HBV-related HCC patients. No significant differences were observed in the genotype distributions of this polymorphism between the patient and control groups. Logistic regression analyses adjusted for age, sex, smoking, and alcohol consumption status revealed that the −607C/A AC and CC genotypes were not associated with HCC risk compared with the AA genotype (*P* = 0.799 and *P* = 0.943). Similar results were observed when comparing the C allele carriers with the reference A allele carriers (*P* = 0.968).

**Table 3:** The genotype and allele frequencies of *IL*-*18* gene polymorphisms between HBV-HCC patients and controls

Polymorphisms	Cases, n = 153 (%)	Controls, n = 165 (%)	χ^2a^	*P* value^a^	OR^b^	95% CI^b^	*P* value^b^
−607C/A							
AA	37 (24.2)	41 (24.8)	0.088	0.957	1.0^ref^	1.0^ref^	
AC	73 (47.7)	76 (46.1)			1.075	0.615–1.881	0.799
CC	43 (28.1)	48 (29.1)			1.024	0.534–1.963	0.943
A allele	147 (48.0)	158 (47.9)	0.957	0.968	1.0^ref^	1.0^ref^	
C allele	159 (52.0)	172 (52.1)			1.006	0.737–1.374	0.968
P-_HWE_	0.584	0.322					
−137G/C							
GG	122 (79.7)	106 (64.3)	9.468	0.008*	1.0^ref^	1.0^ref^	
GC	28 (18.3)	54 (32.7)			0.506	0.290–0.882	0.016
CC	3 (2.0)	5 (3.0)			0.813	0.173–3.802	0.792
G allele	272 (88.9)	266 (80.6)	8.357	0.004*	1.0^ref^	1.0^ref^	
C allele	34 (11.1)	64 (19.4)			0.520	0.332–0.814	0.004*
P-_HWE_	0.363	0.548					

* P < 0.05.

^a^Values about genotype distribution.

^b^Adjusted by age, sex, smoking, and alcohol consumption status.

### Association between −137G/C SNP and HBV-related HCC risk

The genotype and allele frequencies of the *IL*-*18* −137G/C polymorphism among the patient and healthy subject groups are presented in Table 3. The goodness-of-fit χ^2^ test revealed that the genotype frequencies of this SNP in controls were also consistent with the HWE (*P* = 0.548). The frequencies of the GG, GC, and CC were 64.3, 32.7, and 3.0%, respectively, in healthy controls and 79.7, 18.3, and 2.0%, respectively, in HBV-related HCC patients. Significant differences in the genotype distributions of this polymorphism among patients with HBV-related HCC and control subjects were observed (*P* = 0.008). Further, logistic regression analyses adjusted for age, sex, smoking, and alcohol consumption status found that the GC genotype was significantly associated with HCC risk compared with the GG genotype (OR: 0.506, 95% CI: 0.290–0.882, *P* = 0.016). With regards to the −137G/C SNP alleles, significant differences in the C allele distribution were noted when compared with the reference G allele (*P* = 0.004); logistic regression analyses revealed that the C allele was associated with a reduced risk of HBV-related HCC (OR 0.520, 95% CI 0.332–0.814, *P* = 0.004; Table 3).

### Haplotype analyses of *IL*-*18* SNPs and HCC risk

Haplotype analyses were further performed in HBV-related HCC patients and healthy controls using the SHEsis software, in order to evaluate the haplotype frequencies of polymorphisms located in the same chromosome regions and to derive haplotypes specifically correlated with HCC. Four possible haplotypes (A^−607^C^−137^, A^−607^G^−137^, C^−607^C^−137^, C^−607^G^−137^) were derived from the observed genotypes; their distributions in both groups are shown in Table 4. The results showed that the major C^−607^G^−137^ haplotype appeared in 48.4% and 47.0% of cases in the patient and control groups, respectively. Using haplotype analyses, the A^−607^C^−137^ haplotype was observed to be associated with a significantly decreased risk of HCC (OR 0.495; 95% CI 0.294–0.834; *P* = 0.007). The remaining haplotypes were not associated with HCC risk.

**Table 4:** Haplotype frequencies of *IL*-*18* polymorphisms in HCC patients and controls

Haplotypes	Cases (2n = 306, %)	Controls (2n = 330, %)	*P*	OR	95% CI
A^−607^C^−137^	23 (7.5)	47 (14.2)	0.007*	0.495	0.294–0.834
A^−607^G^−137^	124 (40.5)	111 (33.6)	0.073	1.343	0.972–1.856
C^−607^C^−137^	11 (3.6)	17 (5.2)	0.324	0.674	0.306–1.483
C^−607^G^−137^	148 (48.4)	155 (47.0)	0.720	1.059	0.775–1.445

* P < 0.05.

### Association between *IL*-*18* gene polymorphisms and serum IL-18 levels

Serum IL-18 levels showed a markedly skewed distribution and were therefore expressed as the median ± interquartile range (Table 5). Overall, the IL-18 concentration was significantly lower in HBV-related HCC patients (n = 153, 74.40 ± 45.20 ng/L) compared to the controls (n = 165, 112.48 ± 68.65 ng/L; *P* < 0.001). When comparing the difference in serum IL-18 levels between the two groups among individuals with the same genotype, a similar situation was found in which IL-18 levels in HBV-related HCC patients with the -607C/A AA, AC, and CC genotypes and −137G/C GG and GC genotypes were all significantly lower than in healthy control subjects (all *P* < 0.05). No significant difference was observed in the −137G/C SNP CC genotype (*P* = 0.549), but this was mainly attributed to the limited number of subjects with this genotype (n = 3 and n = 5 in cases and controls, respectively). With respect to the difference in serum IL-18 levels among different genotypes within the same group, null significant results were found in both case and control groups, indicating that the −607C/A and −137G/C polymorphisms in the *IL*-*18* gene do not play a pivotal role in the expression of IL-18. Correlation analyses were further conducted to assess the associations between IL-18 levels with alanine aminotransferase (ALT) and aspartate aminotransferase (AST) in both patients and controls, similarly, insignificant results were found (patients: p = 0.810 and p = 0.909, respectively; controls: p = 0.460 and p = 0.304, respectively).

**Table 5:** The association between *IL*-*18* gene polymorphisms and serum IL-18 levels (median ± interquartile range, ng/L) in HCC patients

Groups	Overall	−607C/A (rs1946518)				−137G/C (rs187238)			
		AA	AC	CC	*P* value^a^	GG	GC	CC	*P* value^a^
Controls (n = 165)	112.48 ± 68.65	108.63 ± 105.14 (41)	107.49 ± 71.25 (76)	118.54 ± 46.25 (48)	0.514	118.54 ± 66.47 (106)	101.18 ± 63.20 (54)	108.06 ± 99.24 (5)	0.186
HCC (n = 153)	74.40 ± 45.20	77.48 ± 50.58 (37)	76.33 ± 42.60 (73)	67.10 ± 43.85 (43)	0.202	74.69 ± 47.75 (128)	69.21 ± 36.3 (28)	71.33 ± 52.89 (3)	0.942
*P* value^b^	<0.000	0.002	<0.000	<0.000		<0.000	<0.000	0.549	

^a^Kruskal–Wallis test: comparing the difference of serum IL-18 levels in different genotypes among the same group subjects.

^b^Mann–Whitney U-test: comparing the difference of serum IL-18 levels between the two group subjects among individuals with the same genotype.

## Discussion

HBV infection remains a public health problem worldwide, being endemic in some regions of the world, especially in developing countries [36]. HBV can lead to severe liver diseases such as chronic hepatitis, cirrhosis, and HCC [37]. HBV-infected individuals are at a higher risk of developing HCC [38], and age, sex, alcohol consumption, and smoking status are well known risk factors contributing to HCC incidence [39, 40]. However, these factors do not fully account for the underlying mechanisms in HCC development, indicating that the immune status of individuals may play pivotal roles in disease progression. Through its involvement in the pro-inflammatory cytokine network, *IL*-*18* is an important mediator of innate and adaptive immunity. Polymorphisms of this gene have been reported to influence the expression of IL-18 and may further lead to an alteration in an individual’s immune status, thus increasing carriers’ susceptibility to HCC development. However, this hypothesis is only partially confirmed herein.

In the present study, the *IL*-*18* −137G/C polymorphism was observed to be significantly associated with HCC. The GC genotype and C allele of this SNP were associated with a significantly decreased risk of HCC compared with the GG genotype and G allele. Similarly, the A^−607^C^−137^ haplotype was significantly associated with a lower risk of HCC. Our findings were consistent with those of previous studies on *IL*-*18* polymorphisms and HCC risk [32–34], all of which observed a significant relationship between the −137G/C polymorphism and HCC risk, with a high G allele frequency being associated with an increased risk of HCC and, conversely, a low C allele frequency being associated with a decreased risk of HCC. However, they also found a significantly increased frequency of the −607 C/A AA genotype in HCC patients (AC genotype in Kim et al.’s study [32] ), while we observed a null association between them.

These controversial results may due to the underlying genetic differences between various populations. In a study investigating the relationship between HBV infection and *IL*-*18* promoter polymorphisms among three minority populations in Yunnan province, China, the genotype and allele frequencies of all three SNPs in the *IL*-*18* promoter (−607 C/A, −137G/C, and −656G/T) were found to have distinct distributions [41]. Further, the authors observed a significant difference in HBV infection among the three minority populations, concluding that the difference in genetic background among the various ethnicities may be an important factor responsible for HBV infection susceptibility [41]. The present study was carried out in the Guangxi district, which is also ethnically diverse, and our study population contains both Han and Zhuang ethnicity—this may be the major contributing factor to the inconsistency result found in −607C/A polymorphism.

With regard to the serum IL-18 levels, they were found significantly lower in HCC patients compared to healthy subjects in the present study, such result is controversial to many studies. For instance, researches by Tangkijvanich et al. [42] and Mohran et al. [43] showed that IL-18 levels in HCC patients were significant higher than those in healthy controls, the latter study further concluded that serum IL-18 level was a suitable diagnostic marker in HCV-related HCC patients. However, though Wen et al. [44] also observed significant differences in the transcription and expression levels of IL-18 among different HBV infector groups; interestingly, the highest was found in the fulminant hepatitis group and the lowest in the asymptomatic carrier group, and no significant differences between the chronic hepatitis and normal control groups were observed. These results indicate that serum IL-18 levels may be distinct at different disease stages. On the other hand, as all patients in our study were HBV-related HCC, with hepatitis B surface antigen, HBV core antibody, hepatitis Be antigen, or hepatitis Be antibody seropositive for at least 6 months, the majority of them have gone through antiviral treatment; but, according to He et al. [45], antiviral treatment (pegylated interferon alpha) can induce a marked decline in IL-18 and remission of hepatic inflammatory in HCV infectors. These may be the underlying factors for the inconsistent results observed herein.

Furthermore, no association between the two *IL*-*18* SNPs and IL-18 serum levels were observed in our study, which is inconsistent with previous reports. In the study conducted by Giedraitis et al. [23], three alleles from the *IL*-*18* promoter region (at positions −656, −607, and −137) were cloned and transfected into a HeLa cell line, leading to a tenfold higher *IL*-*18* gene fragment activity compared to the negative control. This indicated that all three polymorphisms had a clear promoting activity and were able to influence the expression of IL-18. However, Giedraitis et al. [23] only conducted studies on gene expression level, which were not fully representative of the transcription levels and, thus, of secretion levels. Therefore, our study is the first to show that −607 and −137 polymorphisms are not associated with the expression of IL-18 in serum. Nevertheless, since serum IL-18 levels may be affected by various factors, some of these may mask the gene polymorphism effects.

In summary, the −137G/C polymorphism of the *IL*-*18* gene was observed to be significantly correlated with HCC risk. The A^−607^C^−137^ haplotype of the −607C/A and −137G/C SNPs was also associated with a decreased risk of HCC. These results indicate that the −137G/C SNP in *IL*-*18* may be a protective factor against HCC. Further, lower serum IL-18 levels were found in HCC patients, however, the *IL*-*18* −607C/A and −137G/C polymorphisms were not associated with IL-18 serum concentration. Considering the limited study population included herein, additional studies with larger samples and detailed clinical data are warranted in various ethnic populations.

## Abbreviations

IL: interleukin
HCC: hepatocellular carcinoma
HBV: hepatitis B virus
IFN-γ: interferon-γ
SNPs: single nucleotide polymorphisms
PCR: polymerase chain reaction
HWE: Hardy–Weinberg equilibrium
ORs: odds ratios
CIs: confidence intervals
SD: standard deviations
